# Discovering drugs to overcome chemoresistance in ovarian cancers based on the cancer genome atlas tumor transcriptome profile

**DOI:** 10.18632/oncotarget.22870

**Published:** 2017-12-04

**Authors:** Fan Wang, Jeremy T-H. Chang, Zhenyu Zhang, Gladys Morrison, Aritro Nath, Steven Bhutra, Rong Stephanie Huang

**Affiliations:** ^1^ Section of Hematology/Oncology, Department of Medicine, University of Chicago, Chicago, IL, USA; ^2^ Biological Sciences Collegiate Division, University of Chicago, Chicago, IL, USA; ^3^ Center for Data Intensive Science, University of Chicago, Chicago, IL, USA; ^4^ Department of Experimental and Clinical Pharmacology, University of Minnesota, Minneapolis, MN, USA

**Keywords:** ovarian cancer, chemoresistance, drug repurposing, TCGA, pharmacogenomics

## Abstract

Ovarian cancer accounts for the highest mortality among gynecologic cancers, mainly due to intrinsic or acquired chemoresistance. While mechanistic-based methods have been used to identify compounds that can overcome chemoresistance, an effective comprehensive drug screening has yet to be developed. We applied a transcriptome based drug sensitivity prediction method, to the Cancer Genome Atlas (TCGA) ovarian cancer dataset to impute patient tumor response to over 100 different drugs. By stratifying patients based on their predicted response to standard of care (SOC) chemotherapy, we identified drugs that are likely more sensitive in SOC resistant ovarian tumors. Five drugs (ABT-888, BIBW2992, gefitinib, AZD6244 and lenalidomide) exhibit higher efficacy in SOC resistant ovarian tumors when multi-platform of transcriptome profiling methods were employed. Additional *in vitro* and clinical sample validations were carried out and verified the effectiveness of these agents. Our candidate drugs hold great potential to improve clinical outcome of chemoresistant ovarian cancer.

## INTRODUCTION

Ovarian cancer is the leading cause of death among gynecological cancers with an average 5-year survival rate of only 46% [1]. The standard of care (SOC) for ovarian cancer is surgery followed by chemotherapy with a combination of a platinum agent (carboplatin or cisplatin) and a taxane (paclitaxel or docetaxel). Even though the majority (about 80%) of ovarian cancer patients respond to the initial chemotherapy, around 20% patients fail to respond. In addition, more than half of initial responders relapse within 3 to 5 years [2, 3]. Therefore, the identification and development of effective drugs against chemoresistant ovarian tumors is of great importance.

Several compounds have been examined to overcome chemoresistance in ovarian cancer based on known biology [4–8]. In addition, high throughput drug screening has been conducted in large variety of cancer cell lines [9–11]. However, efforts to adapt the high throughput drug screening results in order to overcome ovarian chemoresistance have not been reported. To this end, our lab has previously developed pRRophetic, a transcriptome based drug sensitivity prediction tool, which relates cell line drug sensitivity screening datasets with the corresponding cell line transcriptome data to predict *in vivo* drug IC_50_s with great accuracy [12, 13].

In this study, to leverage the predictive power of pRRophetic, we applied it to impute drug sensitivity in the Cancer Genome Atlas (TCGA) ovarian cancer dataset. The rich molecular profiles available in over 500 high-grade serous ovarian cancer (HGSOC) made TCGA ovarian cancer dataset an optimal dataset to comprehensively examine the molecular landscape of ovarian cancer. However, one drawback of the TCGA data is the lack of clearly reported drug sensitivity data. Our work therefore filled this gap by applying drug prediction methods to TCGA in order to generate predicted drug IC_50_ for every ovarian tumor sample.

More importantly, given that both the SOC and drugs that have never been used in treating ovarian cancer have been screened *in vitro*, this allowed us to generate predicted *in vivo* drug sensitivity to a wide range of drugs. By stratifying patients based on their likelihood of responding to SOC chemotherapy, we revealed several drugs that can be more efficacious in tumors that are resistant to SOC. Additionally, *in vitro* and in independent clinical sample validations were carried out to confirm the role of these agents.

## RESULTS

### Predicting drug sensitivities in ovarian tumors based on their transcriptome profiles

Using pRRophetic, we generated 1,773 predicted drug IC_50s_ for all tumors in TCGA ovarian cancer datasets (see Methods; 138 drugs × 598 unique tumor samples). Separate predictions were generated using each of the 4 different transcriptome profiling platforms, including 520 samples for Affymetrix microarray, 574 samples for Agilent microarray, 413 for RNA-Seq, and 266 for RNA-Seq V2 (samples were overlapped among 4 platforms). A high predicted drug IC_50_ represented less sensitive/potential resistance, and conversely a low predicted drug IC_50_ suggested sensitivity.

**Figure 1:**
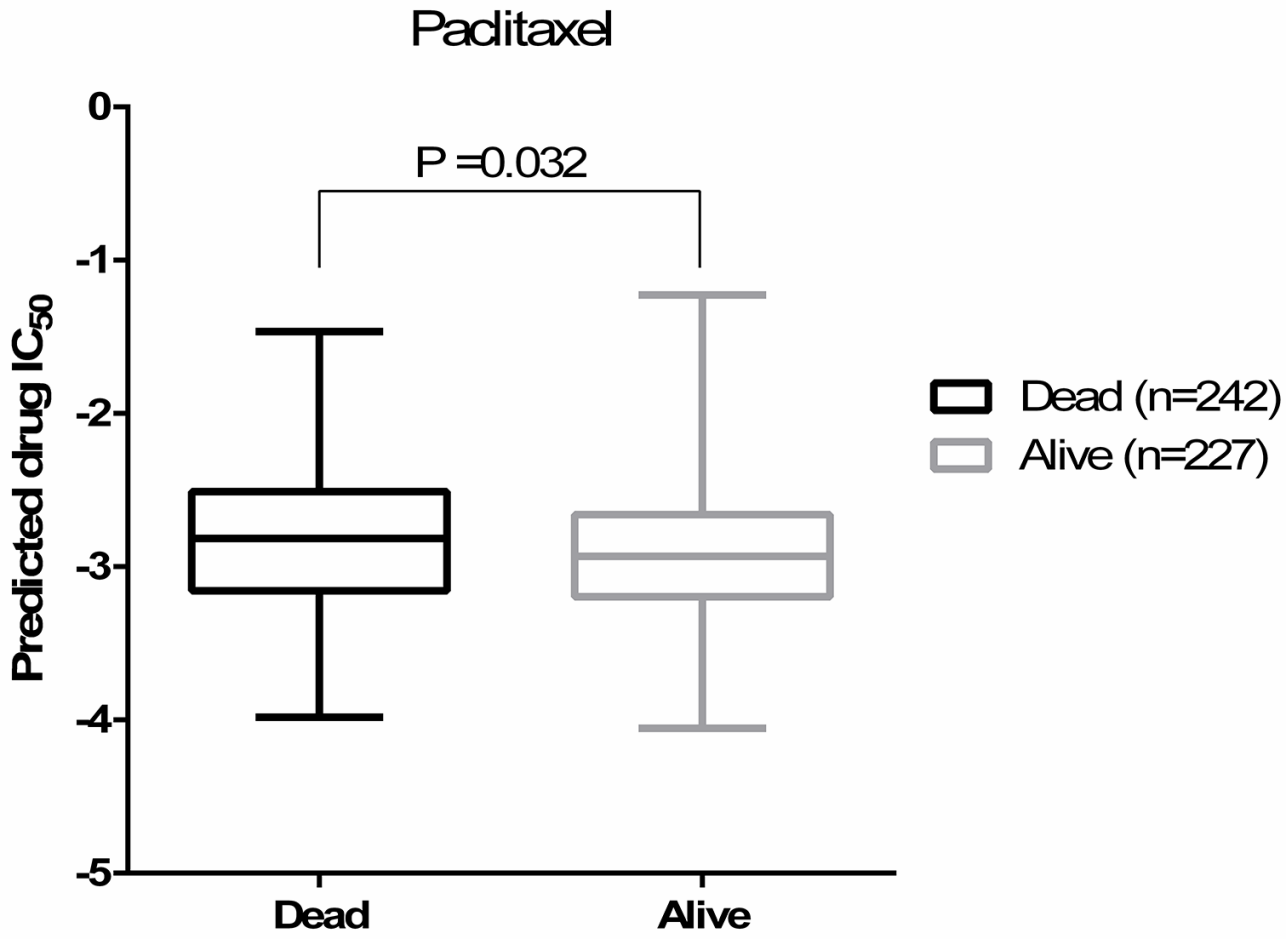
Predicted paclitaxel IC_50_s are correlated with the patients’ survival outcomes (Student’s *t*-test P=0.032) Predicted drug IC_50_ is lower (more sensitive to paclitaxel) in alive group.

As a proof-of-concept, we compared our predicted drug IC_50_s to the patient outcome data (survival) available through TCGA. Here, because of the lack of drug treatment response reported in TCGA, the survival data was used as a surrogate for the measured drug response phenotype. When evaluating predicted vs. actual drug sensitivity (quantified as alive or dead after a given treatment), we observed that in the ovarian cancer patients who were treated with paclitaxel, the predicted drug IC_50_s for paclitaxel were correlated with the patients’ survival outcomes (Figure 1, Student’s *t*-test P=0.032, Wilcoxon rank-sum test P<0.0001). When a tumor is predicted to be more sensitive to the drug (i.e., a lower predicted IC_50_ values), the patient is more likely to be alive. These correlation trends between predicted and observed drug sensitivity were also observed for docetaxel and cisplatin, although not statistically significant. Note that only a subset of TCGA ovarian cancer samples contain treatment information with the highest numbers of patients treated with paclitaxel (n=469). Given ovarian cancer survival is highly correlated with disease stages, we also fitted a regression model between survival and predicted paclitaxel sensitivity controlling for disease stage. Once again, we observed a significant correlation between the predicted paclitaxel IC_50_ and the survival outcomes of those patients underwent paclitaxel treatment (P=0.0385). Only 112 and 154 patients were known to be treated with docetaxel and cisplatin, respectively, suggesting that we may be underpowered to observe such an association for these other drugs.

### Identify drug candidates that may work in the SOC resistant ovarian tumors

**Figure 2:**
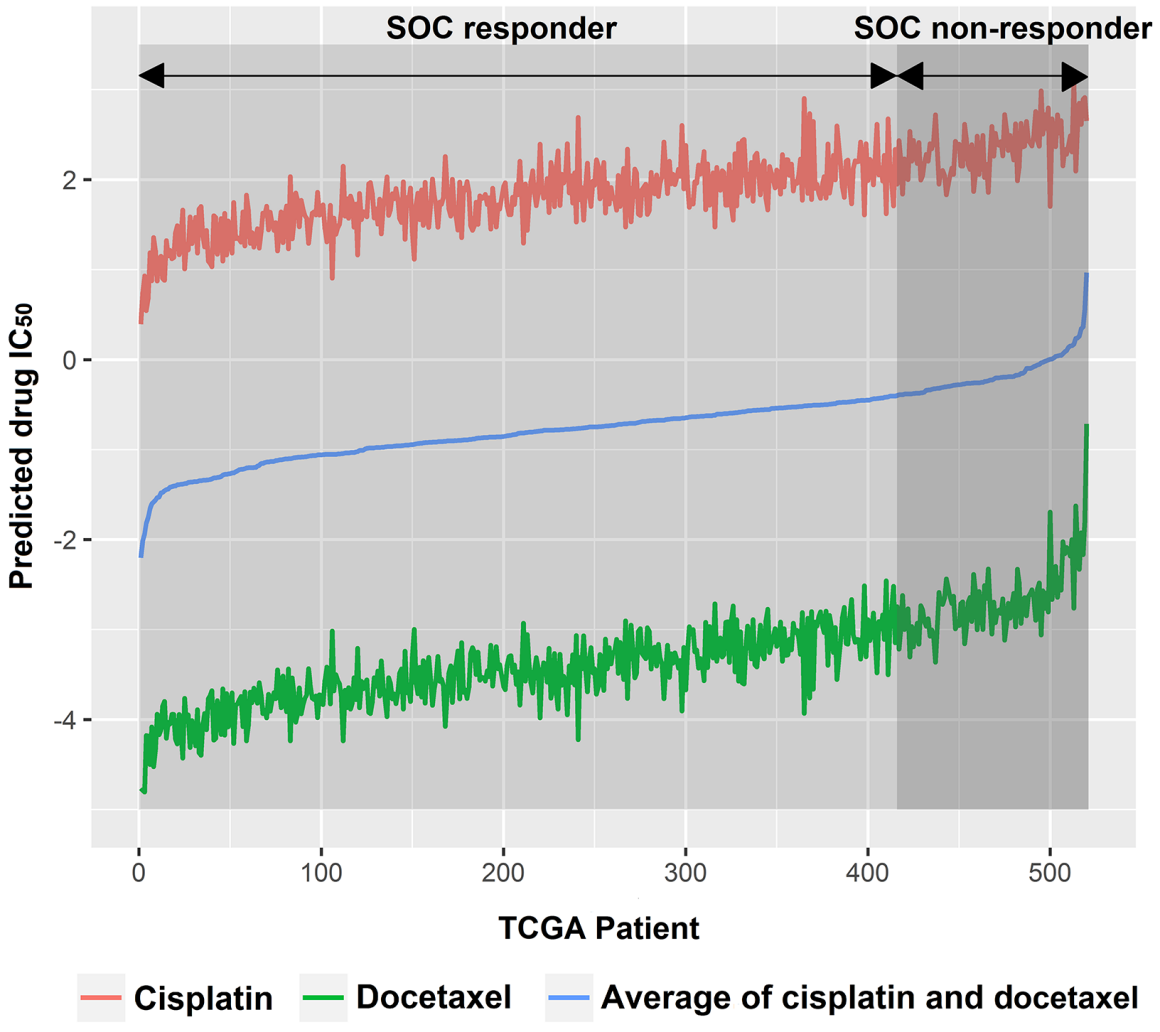
TCGA ovarian cancer patients were subgrouped into SOC responders and SOC non-responders SOC, standard of care.

For every tumor sample, we averaged the predicted drug IC_50_ of cisplatin and docetaxel, the current SOC, as an indicator of individual tumor sensitivity to SOC (See blue line in Figure 2). Given 80% SOC response rate was well documented by literature [2, 3], we stratified the 20% tumor with higher predicted SOC IC_50_ as SOC non-responders (Figure 2). We then performed a Student’s *t*-test for predicted drug IC_50_ for all other drugs between SOC responders and non-responders. Only those drugs that showed lower predicted IC_50_ in SOC non-responders groups than SOC responders (P<0.05) were further evaluated.

**Figure 3:**
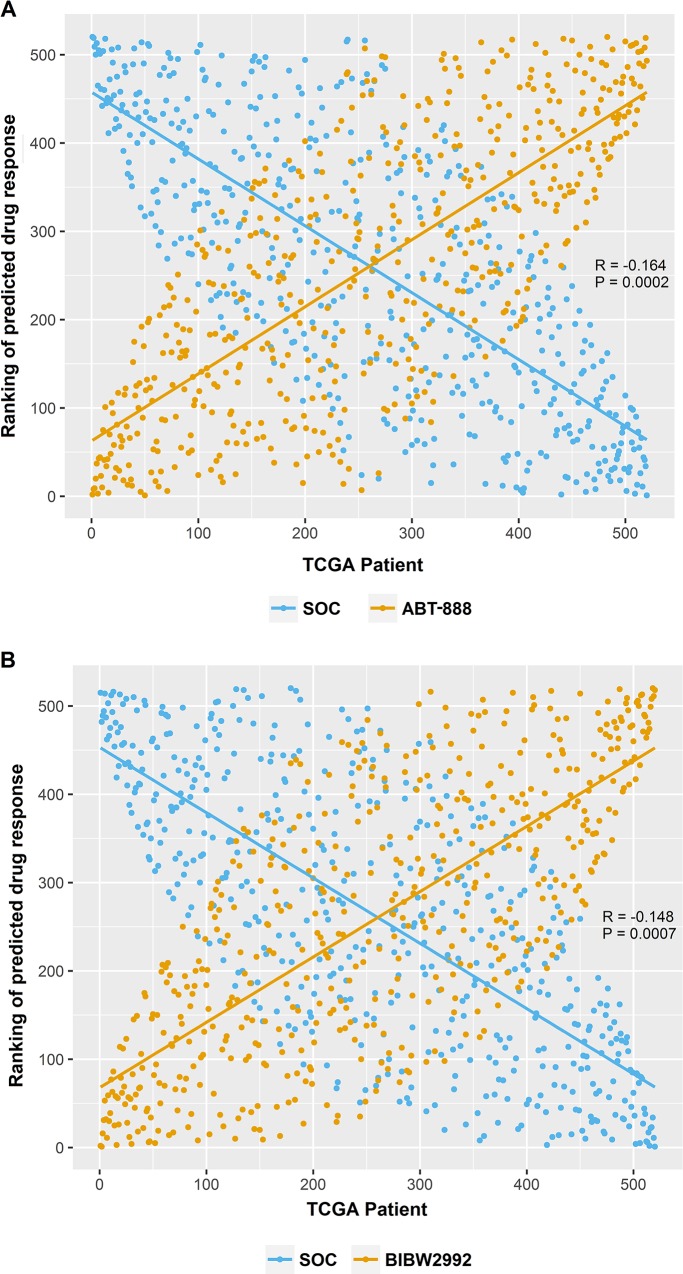
The opposite effect patterns between candidate drugs and SOC **(A)** Significant negative Pearson correlation between the ranking of SOC and ABT-888 (R_p_= −0.164, P_p_= 0.0002). **(B)** Significant negative Pearson correlation between the ranking of SOC and BIBW2992 (R_p_= −0.148, P_p_= 0.0007).

Each of the 4 transcriptome profiles for these samples were analyzed separately to generate predicted drug IC_50_s. Therefore, four sets of candidate drugs were identified using each transcriptome profiling dataset. Specifically, we identified 13, 17, 18 and 12 drugs with the Affymetrix arrays, Agilent arrays, RNA-Seq, and RNA-Seq V2 datasets, respectively. (Details about these drug candidates identified from each platform can be found in Supplementary Table 1). Among them, 5 drugs were identified in all four datasets. They were ABT-888, BIBW2992, gefitinib, AZD6244, and lenalidomide (Table 1). For each candidate drug, the average predicted drug IC_50_ in SOC sensitive and resistant group were reported in Table 1. All 5 candidate drugs had significantly lower predicted drug IC_50_ (suggesting more sensitive) in the SOC resistant group than those in SOC sensitive group (P<0.05, two-tailed Student’s *t*-test). Significant negative Pearson correlations were shown in Figure 3 between ABT-888 (R= −0.164, P= 0.0002), or BIBW2992 (R= −0.148, P= 0.0007), and SOC when rank order patients based on their predicted sensitivity to these drugs. To ensure the robustness of the results, we also performed Pearson test using the actual predicted drug IC_50_. Again, significant negative correlations were shown between SOC and ABT-888 (R= −0.222, P<0.0001), or BIBW2992 (R= −0.412, P<0.0001). These inverse correlations for drug response indicated that our candidate drugs were predicted to be more efficacious in SOC resistant patients. Notably, the average predicted drug IC_50_ of each given drug in the SOC resistant or sensitive groups were very close between different datasets. This suggests that our prediction was highly reproducible even though different technologies were applied to obtain gene expression profiles.

**Table 1:** Summary of the predicted drug IC_50_ for candidate drugs in SOC responders and non-responders analyzed using different expression profiling platforms

	TCGA discovery datasets												Validation dataset		
	Affymetrix		Agilent				RNA-Seq			RNA-Seq V2			CTRP v2		
	Predicted drug IC_50_		Student’s *t*-test P value	Predicted drug IC_50_		Student’s *t*-test P value	Predicted drug IC_50_		Student’s *t*-test P value	Predicted drug IC_50_		Student’s *t*-test P value	P value *^a^*	Correlation analysis *^b^*	
	SOC Res-ponders	SOC Non-responders		SOC Res-ponders	SOC Non-responders		SOC Res-ponders	SOC Non-responders		SOC Res-ponders	SOC Non-responders			Spearman	Pearson
**ABT-888**	5.35	5.27	3.17×10^−5^	5.34	5.30	7.69×10^−3^	5.35	5.26	1.26×10^−4^	5.35	5.25	1.17×10^−3^	0.011	R= −0.119, P= 0.023	R= −0.112, P= 0.029
**BIBW2992**	2.27	2.14	7.95×10^−4^	2.28	2.18	2.63×10^−11^	2.28	2.19	1.82×10^−7^	2.28	2.19	3.57×10^−5^	0.031	R= −0.318, P< 0.0001	R= −0.276, P< 0.0001
**Gefitinib**	2.05	1.79	8.92×10^−8^	2.04	1.84	1.00×10^−6^	2.03	1.90	6.62×10^−3^	2.05	1.85	1.02×10^−3^	0.383	R= −0.302, P< 0.0001	R= −0.273, P< 0.0001
**AZD6244**	3.05	2.73	2.73×10^−6^	3.04	2.80	3.74×10^−4^	3.06	2.72	7.44×10^−6^	3.07	2.68	7.77×10^−5^	0.338	R= −0.272, P< 0.0001	R= −0.259, P< 0.0001
**Lenalidomide**	5.40	5.30	2.71×10^−8^	5.39	5.35	1.57×10^−3^	5.39	5.32	1.04×10^−4^	5.39	5.33	8.82×10^−3^	NA	R= −0.142, P=0.008	R= −0.139, P=0.009

*^a^* P value was calculated from Student’s *t*-test by comparing Predicted drug IC_50_ between SOC responders and non-responders.

*^b^* Correlation analysis was performed between predicted SOC IC_50_ and candidate drug IC_50._

### Validation

For validation, we applied the same methods to an independent ovarian cancer dataset: the Australian Ovarian Cancer Study (AOCS, n=285). Significant higher predicted sensitivity (lower predicted IC_50_) in SOC resistant ovarian tumors were confirmed for BIBW2992 (P=0.003) using 80/20 (responder vs. non-responder) cutoff. To test if the results were robust to the choice of cutoff, we stratified SOC sensitivity using the 50/50 predicted SOC IC_50_ as threshold (50% sensitive and 50% resistant) as well. As a result, all candidate drugs— AZD6244, gefitinib, BIBW2992, lenalidomide and ABT-888, were significantly more sensitive in the SOC resistant tumors (P<0.05). In addition, we performed correlation analysis between predicted SOC and candidate drug IC_50_. Significant correlations were found for all drugs as ABT-888 (R_s_= −0.119, P_s_= 0.023, R_p_= −0.112, P_p_= 0.029), BIBW2992 (R_s_= −0.318, P_s_< 0.0001, R_p_= −0.276, P_p_< 0.0001), gefitinib (R_s_= −0.302, P_s_< 0.0001, R_p_= −0.273, P_p_< 0.0001), AZD6244 (R_s_= −0.272, P_s_< 0.0001, R_p_= −0.259, P_p_< 0.0001), and lenalidomide (R_s_= −0.142, P_s_=0.008, R_p_= −0.139, P_p_=0.009).

**Figure 4:**
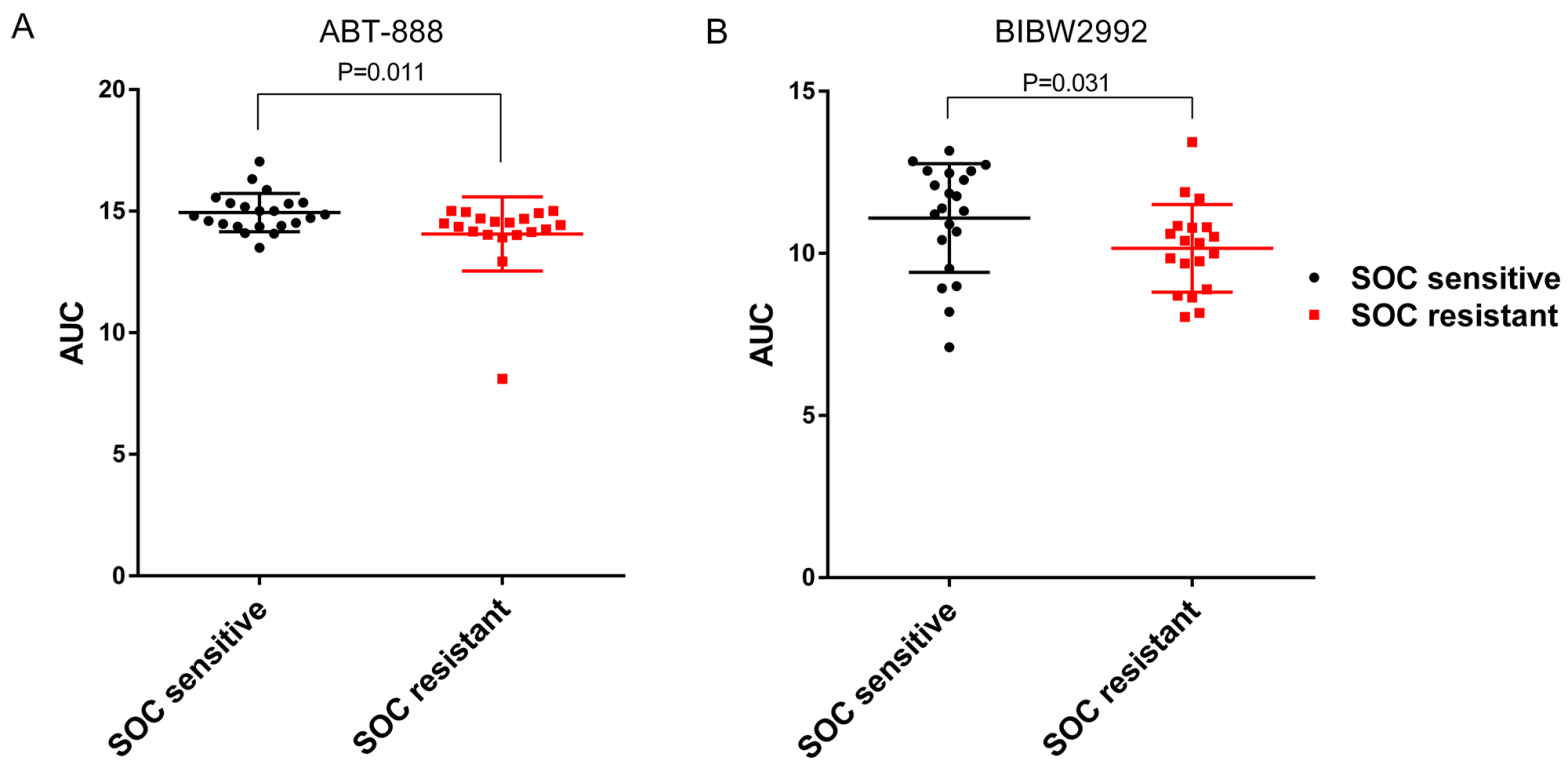
ABT-888 and BIBW2992 are more sensitive in SOC resistant ovarian cancer cell lines tested in CTRP v2 *In vitro* measured drug sensitivities (AUC) in CTRP v2 are compared between SOC sensitive and resistant cell lines. The higher the AUC, the more resistance the cell line has for a given drug. **(A)** ABT-888 showed significant lower AUC in SOC resistant group (Student’s *t*-test P=0.011). **(B)** BIBW2992 showed significant lower AUC in SOC resistant group (Student’s *t*-test P=0.031).

For *in vitro* validation, we employed a large scale independent cell line drug sensitivity screening data set – CTRP v2 [14]. In CTRP v2, 4 of the 5 candidate drugs were screened (ABT-888, BIBW2992, gefitinib and AZD6244). The AUCs (area under the dose response curve) were used to define cellular sensitivity to these drugs. Given the small sample size (41 ovarian cancer cell lines in total) in CTRP v2, we stratified 22 cell lines as sensitive to SOC and 19 as resistant based on mean predicted SOC IC_50_. Then, for the four drugs, differences of AUCs between SOC resistant and sensitive group were analyzed using one-tailed t-test. P-values were presented in Table 1. ABT-888 and BIBW2992 showed significant lower AUC (suggesting higher sensitivity) in SOC resistant group (Figure 4A, P=0.011 for ABT-888; Figure 4B, P=0.031 for BIBW2992). There was no significant difference between the SOC resistant and sensitive groups (P>0.05) for gefitinib and AZD6244.

### Identification of pathways associated with the SOC resistance and candidate drugs sensitivity

To further explore the underlying biology that leads to the effectiveness of the 5 candidate drugs in SOC resistant ovarian tumors, we performed Gene Set Enrichment Analysis (GSEA) using the KEGG pathway gene sets. The pathways listed in the left column of Table 2, were statistically significant positive correlation to SOC sensitivity and negative correlation to sensitivities of the candidate drugs (FDR q-value <0.25). In other words, the enrichment of KEGG pathways we selected could make the tumors more resistant to SOC, at the same time more sensitive to candidate drugs.

**Table 2:** Summary of enriched KEGG pathways that were significantly correlated with resistance of SOC and sensitiveness of candidate drugs

	Positively correlated (desensitize tumor to drug)	Negatively correlated (sensitize tumor to drug)					
	SOC	ABT-888	BIBW2992	Gefitinib	AZD6244	Lenalidomide	
Allograft rejection	0.45	−0.861	−0.808	−0.797	−0.755	−0.602	
Graft versus host disease	0.4	−0.847	−0.827	−0.808	−0.761	−0.542	
Type I diabetes mellitus	0.389	−0.803	−0.736	−0.774	−0.708	−0.515	
Antigen processing and presentation	0.372	−0.736	−0.68	−0.683	−0.63	−0.453	
RIG I like receptor signaling pathway	0.364	−0.597	−0.543	−0.453	−0.521	−0.235	
Autoimmune thyroid disease	0.354	−0.819	−0.689	−0.742	−0.695	−0.517	
Apoptosis	0.311	−0.517	−0.519	−0.488	−0.503	−0.185	
Asthma	0.302	−0.79	−0.72	−0.767	−0.715	−0.414	
Intestinal immune network for IGA production	0.291	−0.795	−0.633	−0.742	−0.66	−0.575	
TOLL like receptor signaling pathway	0.27	−0.626	−0.539	−0.549	−0.585	−0.22	
Leishmania infection	0.26	−0.712	−0.614	−0.7	−0.661	−0.223	
NOD like receptor signaling pathway	0.232	−0.677	−0.565	−0.586	−0.631	−0.29	
Cytosolic DNA sensing pathway	0.216	−0.686	−0.576	−0.538	−0.588	−0.31	
Natural killer cell mediated cytotoxicity	0.159	−0.668	−0.515	−0.622	−0.611	−0.404	

The numbers in this table indicated the Enrichment Score (ESs).

Interestingly, among the pathways that were enriched for SOC resistance and candidate drug sensitivity (Table 2), were the apoptosis and cytosolic DNA sensing pathway. Both apoptosis and cytosolic DNA sensing pathways desensitized tumors to SOC, which was supported by literature [15]. ABT-888, a PARP1/2 inhibitor, plays a role in inhibition of DNA repair and introduction of cell death. Gefitinib and BIBW2992 are both EGFR inhibitors, which could induce apoptosis by inhibition of Ras signaling [16].

## DISCUSSION

Taxane and platinum based chemotherapy was introduced 20 years ago to treat ovarian cancer as standard first line regimen. There has been little progress in the last decade to improve the overall survival for chemoresistant ovarian cancer patients. In this study, we applied a novel transcriptome-based drug sensitivity prediction method to a collection of large *in vivo* and *in vitro* ovarian cancer datasets. Importantly, we identified five agents—ABT-888, BIBW2992, gefitinib, AZD6244, and lenalidomide that exhibit higher sensitivity in SOC resistant ovarian cancers in multi-platform TCGA datasets. We provided further validation of these drugs’ sensitivity in additional clinical samples derived from ovarian cancer patients (through AOCS) and *in vitro* (through CTRP v2).

Although survival information has been collected for almost all TCGA samples, the treatment information for each patient is lot sparse, ranging from 40-97% coverage for each disease type to date. Our work bypassed this issue by generating our own *in vivo* drug sensitivity prediction. This method has been tested previously *in silico* analysis of 3 independent clinical trials and has demonstrated superior power [12]. Furthermore, in our proof-of-concept analysis, we observed a significant different predicted drug IC_50_s between dead and alive patients who were treated with paclitaxel. It is ideal to use response to treatment as a phenotype when comparing to our predicted drug IC_50_; however, since such data is not present, we opted to examine relationship between predicted drug sensitivity and survival outcome as a surrogate for treatment response.

BIBW2992 (afatinib) is an irreversible tyrosine kinase inhibitor (TKI) that inhibits ErbB family members including EGFR, HER2, and HER4. Thereby BIBW2992 can target downstream oncogenic signaling pathways. The mechanism of its anti-cancer activity has been investigated mainly in non-small cell lung cancers (NSCLC) cells. BIBW2992 induces apoptosis by activating pro-apoptotic autophagy or through Elk-1/CIP2A/PP2A/AKT pathway in NSCLC cells [17]. It was approved by the FDA as first-line treatment of EGFR-mutated NSCLC in 2013. A number of clinical trials for various solid tumors including breast, prostate and head and neck cancer also have reported promising outcomes [18–20]. We are unaware of trials of BIBW2992 conducted in ovarian cancer; however, some pre-clinical results indicated encouraging prospects. For example, Wang SQ et al. demonstrated that BIBW2992 reverses multidrug resistance in ovarian cancer cells by dually inhibiting ATP binding cassette subfamily B member 1 [17]. They found BIBW2992 enhanced the paclitaxel-induced apoptosis *in vitro* and in mouse model, which strongly supports our findings and suggests BIBW2992 may be an effective treatment option for chemoresistant ovarian cancers. Moreover, another group compared multiple HER inhibitors in terms of growth inhibition of ovarian cancer cell lines. Irreversible pan-HER family TKIs including BIBW2992 were more effective than EGFR specific TKIs gefitinib and erlotinib, the dual EGFR/HER-2 TKI lapatinib or the reversible pan EGFR/HER-2/HER-3 TKI sapitinib [21]. Therefore, we propose that BIBW2992 may provide a more effective way to overcome chemoresistance in ovarian tumors.

Impressively, of the 5 drugs we discovered that may be useful in overcoming SOC resistance in ovarian cancer, 4 have had ongoing clinical studies and the preliminary findings suggest the promise/validity of our predictions from pRRophetic. The first *in vitro* validated candidate drug is ABT-888 (veliparib), a potent inhibitor of PARP1 and PARP2. Poly (ADP-ribose) polymerase (PARP) is essential enzyme involved in damaged DNA detection and repair through the base excision repair pathways. The inhibition of PARP can sensitize tumors to cytotoxic agents by blocking DNA repair, followed by cell cycle arrest and apoptosis, and possibly make tumors more susceptible to DNA-damaging agents including carboplatin [22]. PARP inhibitors have shown preclinical activity in cancers that are deficient in DNA repair due to defects in homologous recombination (HR), eg. BRCA-mutated tumors [23, 24]. PARP inhibitors were initially tested in trials as treatment for BRCA mutation-associated ovarian and breast cancers. The TCGA discovery on the defects in the HR pathway commonly existed in more than 50% of high-grade serous ovarian cancers [25], had led to further investigation of PARP inhibitors in a wider population of ovarian cancers. Olaparib, as the first FDA-approved PARP inhibitor, is used to treat recurrent BRCA mutant ovarian cancer patients. ABT-888 is undergoing a number of clinical trials in combination with SOC, and also as maintenance in the first-line treatment of ovarian cancer. In a single-agent Phase I trial [26] designed for platinum-refractory ovarian or basal-like breast cancers, ABT-888 showed higher response rate and clinical benefit rate in BRCA-mutated tumors than BRCA wild-type tumors. Another single-agent Phase II study [27] in BRCA-mutated ovarian cancer patients has also been reported. ABT-888 was well tolerated with a response rate of 26%. It remains unclear whether PARP inhibitors should be utilized as newly-diagnosed or relapsed patients, single agent or in combination with SOC or as maintenance treatment. Further investigations will help to answer the questions and unveil its full potential as a treatment option.

Gefitinib, a selective epidermal growth factor receptor (EGFR) tyrosine kinase inhibitor, can competitively inhibit ATP binding on EGFR. Gefitinib was approved by the FDA as monotherapy treatment for patients with locally advanced or metastatic non-small cell lung cancer (NSCLC) after failure of both platinum and taxane based chemotherapies [28]. EGFR is commonly present in 33% to 75% of ovarian cancers and increased EGFR is associated with poor survival in ovarian cancer patients [29]. A Phase II trial with gefitinib in combination with paclitaxel and carboplatin as a second-line treatment for advanced ovarian adenocarcinoma, showed a high rate of 63% overall response [30]. Our study provides additional supporting evidence in further pursuing the evaluation of gefitinib in SOC resistant ovarian cancer treatment.

Another example is AZD6244 (selumetinib), a potent, highly selective MEK1/2 inhibitor which also inhibits ERK1/2 phosphorylation. It has reported single agent activity in several trials on solid tumor including recurrent low-grade serous ovarian cancer (LGSOC). A recent phase II AZD6244 trial in LGSOC showed better efficacy than SOC in terms of response rate and disease stabilization [31]. One of the reasons to platinum resistance has been proposed as through the activation of JNK and ERK cascades by cisplatin-induced DNA damage, where JNK and ERK are required for cell proliferation and differentiation. The inhibition of JNK or ERK cascades can sensitize ovarian cancer cells to cisplatin [32]. Therefore, it is not surprising that AZD6244, as an ERK1/2 inhibitor, may promote sensitivity to cisplatin. The findings along with our work warrants further investigation of this drug in ovarian cancer.

Lenalidomide is an antiangiogenic agent, with capability as an immunomodulator, which can inhibit hypoxia-inducible factor (HIF)-1α, an essential regulator of metastasis. Lenalidomide was approved by the FDA in 2006 to treat patients with multiple myeloma in combination with dexamethasone. Its clinical efficacy has also been reported as a single agent and combined with chemotherapy in solid tumors, including ovarian, prostate, renal cell and hepatocellular cancers [33, 34]. Most of the Phase I trials on ovarian cancer showed an acceptable safety profile, while one of them was terminated because of toxicity. Further studies of lenalidomide may be warranted in this disease setting.

Several compounds have been proposed for overcoming ovarian cancer chemoresistance, including bortezomib [4], antiprogestin compounds [5], combined treatment with death ligand TRAIL and antidiabetic acting PPARγ ligands [6], P-glycoprotein (P-gp) inhibitors [7, 8], cancer stem cell targeting agents and autophagy based modulation [7]. Most of them, except for bortezomib, were not screened in the anti-cancer drug sensitivity database pRRophetic was built on. Adam et al. observed that bortezomib combined with paclitaxel act in a synergistic manner. There was more than 2-fold decrease in IC_50_ when treating the cells (SKOV3 and A2780) with the combination as compared to paclitaxel alone [4]. Our screen did not reveal the role of bortezomib in SOC resistant ovarian cancer possibly because our work focused on single agent efficacy rather than drug combination.

Our GSEA results indicated that pathways related to apoptosis and cytosolic DNA sensing were important in SOC resistance. These have been supported by various literature [35–37]. For example, enhanced DNA repair [35], induction of anti-apoptotic protein [37] and activation of the AKT [37] have been demonstrated to be the major contributing factors for chemoresistance in ovarian cancer. At the same time, these pathways were found to be enriched in increasing sensitivity to our candidate drugs. ABT-888, as a PARP inhibitor, could block the repair of single-strand DNA breaks and result in accumulation of single-strand breaks and subsequently double-strand breaks. The inability of DNA repair will cause chromosomal instability, cell cycle arrest and ultimately cell death. It is therefore not a surprise that up-regulated DNA repair and apoptosis pathway could decrease the vulnerability of chemotherapy, however, increase the vulnerability of DNA repair targeted ABT-888.

Although the effectiveness of all 5 candidate drugs was reproduced in AOCS, an independent clinical ovarian cancer study, only 2 of the 5 drugs were validated *in vitro* using CTRP v2. The reasons may be 1) lenalidomide was not screened in CTRP v2; and 2) much smaller sample size in the *in vitro* validation dataset when compared to the *in vivo* validation dataset (41 vs 285).

In conclusion, by applying a novel drug sensitivity prediction approach to a set of large *in vivo* and *in vitro* datasets, we discovered and validated several candidate drugs that could be more effective in SOC resistant ovarian cancer patients. GSEA analysis unveiled the pathways that may account for the improved efficacy of our candidate drugs comparing with SOC. Clinical trials evaluating the effectiveness of some of these candidate drugs either alone or in combination with chemotherapy are ongoing in ovarian cancer. They should also be carefully examined in the SOC resistant setting.

## MATERIALS AND METHODS

### Overview

pRRophetic method simultaneously constructs prediction models using transcriptome and drug sensitivity data derived from the Cancer Genome Project (CGP) and apply it to the datasets that contain transcriptome information (i.e., the TCGA) to generate predicted drug IC_50_s.

Given that there are 138 drugs screened in CGP [9], we were able to generate predicted drug IC_50_s of all 138 drugs in each tumor sample. In this study, we generated predicted drug IC_50_s *in vivo* for two independent large ovarian cancer datasets: the TCGA and Australian Ovarian Cancer Study (AOCS) [38]. Furthermore, to validate our prediction, we generated predicted drug IC_50_s in another large *in vitro* cancer cell line drug screening dataset, the Cancer Therapeutics Response Portal v2 (CTRP v2) [14] ; and compared the predicted drug IC_50_ to the experimentally measured cellular sensitivity (eg. AUC) to drugs.

Specifically, TCGA ovarian cancer gene expression profiles were downloaded from TCGA data portal (https://tcga-data.nci.nih.gov/tcga/). There are four sets of transcriptome profiles for ovarian cancer: two generated using microarrays— Affymetrix HT Human Genome U133 and Agilent 244K Custom Gene Expression G4502A-07; and the other two with RNA-seq. The Affymetrix platform contained expression profiles of 520 patients, and Agilent platform contained expression profiles of 574 patients. Illumina HiSeq 2000 RNA Sequencing platform contained 413 patients’ tumor expression profile, and Illumina HiSeq 2000 RNA Sequencing Version 2 has expression profiles for 266 patients. Although the samples from these four expression datasets were highly overlapped, because the different transcriptome profiling technologies have different specificity, sensitivity and dynamic range, we examined each dataset separately as technical replications. Findings from each analysis were compared and only those drugs that predicted to be more sensitive in SOC resistant tumors by all 4 analyses were further evaluated.

Transcriptome data for AOCS was generated using Affymetrix U133_plus2 microarray and obtained through GEO (GSE9891, http://www.ncbi.nlm.nih.gov/geo/query/acc.cgi?acc=GSE9891). This includes gene expression data for 285 ovarian tumor samples.

For prediction validation, we employed CTRP v2 [38], in which area under the dose response curve (AUCs) for 481 compounds were available in 860 cancer cell lines. The drug sensitivity data was downloaded from CTD^2^ DATA PORTAL (https://ctd2.nci.nih.gov/dataPortal/). The mRNA expression (Affymetrix U133+2 array) data of the cell lines was obtained from Cancer Cell Line Encyclopedia (CCLE) data portal (http://www.broadinstitute.org/ccle).

### *In vivo* identification of drugs that may work in SOC resistant ovarian cancer patients

We chose to analyze TCGA ovarian tumors as our discovery dataset because it is one of the largest ovarian cancer datasets where algorithms such as pRRophetic could be employed, while AOCS dataset was used as independent *in vivo* validation dataset. For each tumor sample in TCGA and AOCS, we generated 138 predicted drug IC_50_s. Specific for TCGA, predicted drug IC_50_ was generated independently using each set of the four transcriptome profiling datasets separately. Note that lower predicted drug IC_50_ indicated higher sensitivity to such a drug.

We calculated ovarian cancer patients’ SOC IC_50_ by averaging the predicted drug IC_50_ to cisplatin and docetaxel for every patient. Furthermore, with the commonly observed 80% response rate to SOC in ovarian cancer [3, 4], we classified TCGA ovarian cancer samples as either resistant to SOC (as the top 20% ranked SOC response) or sensitive to SOC (the bottom 80% ranked SOC response).

We then evaluated the predicted drug IC_50_s of other 136 drugs to discover drugs showing opposite predicted efficacy profile to that of SOC using Student’s *t*-test. In another word, we aimed at identifying drugs with lower predicted drug IC_50_ (meaning higher sensitivity) in SOC resistant patients. The same selection method was applied to all 4 TCGA gene expression datasets. The candidate drug lists were overlapped to generate the final candidate drug list. To avoid findings dependent on the arbitrary cutoff used in defining SOC response, in addition to the 80/20 (responder vs. non-responder) cutoff, we also evaluated the 50/50 (responder vs. non-responder) cutoff as well as employing correlation analysis between SOC and candidate drugs. P values less than 0.05 were considered significant.

### *In vivo* and *in vitro* validation of candidate drugs

The findings from TCGA were first validated through the AOCS dataset. Given CTRP v2 has measured hundreds of drugs’ sensitivities in ovarian cancer cell lines (including some of the candidate drugs we predicted to be more sensitive in TCGA and AOCS), we performed separate predictions using CTRP v2 ovarian cancer cell lines. Specifically, gene expression profiles was available in 41 of the 43 ovarian cancer cell lines in CTRP v2. Once again, cell lines sensitivity to SOC were generated by averaging the predicted drug IC_50_ of cisplatin and docetaxel. CTRP v2 measured and reported AUC as drug sensitivity with the higher the AUC, the more resistant the cells to any given drug. Then we performed Student’s *t*-test on candidate drug AUCs between SOC sensitive cell lines and SOC resistant cell lines.

### Gene set enrichment analysis (GSEA)

To further explore the potential mechanism underlying the observed opposite therapeutic effect of our candidate drugs and the SOC, we performed GSEA analysis (GSEA software v2.2.2, www.broadinstitute.org/gsea) using the KEGG pathway gene sets. We input TCGA-Agilent expression dataset and predicted SOC IC_50_s and those of the candidate drugs as phenotypes. The association between drug sensitivities and gene expression was run separately for each phenotype following the developer’s protocol (http://www.broad.mit.edu/gsea/). FDR q-value <0.25 was used to define significantly regulated pathways. The positively SOC-correlated pathways were overlapped with pathways that were significantly and negatively correlated with all candidate drugs.

## SUPPLEMENTARY MATERIALS TABLE



## Abbreviations

TCGA: The Cancer Genome Atlas
SOC: standard of care
CGP: Cancer Genome Project
CTRP v2: Cancer Therapeutics Response Portal v2
AOCS: Australian Ovarian Cancer Study
AUC: area under the dose response curve
CCLE: Cancer Cell Line Encyclopedia
GSEA: Gene Set Enrichment Analysis
