# Supplementary material for: Discovering drugs to overcome chemoresistance in ovarian cancers based on the cancer genome atlas tumor transcriptome profile
Source: Oncotarget. 2017 Dec 4;8(70):115102–13. doi: 10.18632/oncotarget.22870 (PMC5777757; doi:10.18632/oncotarget.22870)
Supplement: Supplementary file 1 [file oncotarget-08-115102-s001.pdf]

Discovering drugs to overcome chemoresistance in ovarian cancers based on the cancer genome atlas tumor transcriptome profile

SUPPLEMENTARY MATERIALS

Supplementary Table 1: Candidate drugs identified in 4 TCGA datasets

Affymetrix	Agilent	RNA-Seq	RNA-Seq V2
**AZD6244**	**AZD6244**	**AZD6244**	**AZD6244**
**Gefitinib**	**Gefitinib**	PD-0325901	PD-0325901
**BIBW2992**	PD-0325901	PAC-1	**Gefitinib**
CI-1040	AKT-inhibitor-VIII	AS601245	CHIR-99021
SB590885	Lapatinib	**Gefitinib**	AS601245
PLX4720	AS601245	Shikonin	Shikonin
Nilotinib	**BIBW2992**	SB590885	**BIBW2992**
**Lenalidomide**	CHIR-99021	FH535	VX-702
VX-702	FH535	AKT-inhibitor-VIII	CI-1040
AKT-inhibitor-VIII	JW-7-52-1	VX-702	**Lenalidomide**
**ABT-888**	Salubrinal	**ABT-888**	Bicalutamide
PF-02341066	**ABT-888**	Nilotinib	**ABT-888**
Roscovitine	**Lenalidomide**	**BIBW2992**	
	Bryostatin-1	**Lenalidomide**	
	Bicalutamide	CI-1040	
	Roscovitine	FTI-277	
	CGP-082996	Bicalutamide	
		Roscovitine	

* Candidate drugs are highlighted in bold.
